# Extreme Intracranial Hypotension With Brain Herniation Treated With Repeat Bolus Intrathecal Infusions

**DOI:** 10.7759/cureus.8089

**Published:** 2020-05-13

**Authors:** Douglas J Chung, Jason Liounakos, Kevin Abrams, Vitaly Siomin

**Affiliations:** 1 Neurological Surgery, Herbert Wertheim College of Medicine, Florida International University, Miami, USA; 2 Neurological Surgery, University of Miami, Miami, USA; 3 Neuroradiology, Baptist Hospital of Miami, Baptist Health South Florida, Miami, USA; 4 Neurological Surgery, Miami Cancer Institute, Baptist Health South Florida, Miami, USA

**Keywords:** intracranial hypotension, intrathecal infusion, brain herniation

## Abstract

Intracranial hypotension (IH) is a relatively common condition associated with low cerebrospinal (CSF) pressure. The most typical symptom is orthostatic headache, although neurological deficits and changes in the level of consciousness, such as encephalopathy, stupor, and coma, may also occur. Uncomplicated CSF hypotension headaches generally resolve with rest, hydration, and analgesia. However, persistent cases may require an epidural blood patch (EBP) for resolution. Our report presents the case of a 50-year-old male with a history of intravenous (IV) drug abuse, positive for human immunodeficiency virus (HIV), hepatitis B virus (HBV), and hepatitis C virus (HCV) antibodies, who was admitted for new-onset headache and brain magnetic resonance imaging (MRI) findings suggesting CSF hypotension. The patient subsequently developed altered mental status with agonizing respirations, prompting intubation and admission to the intensive care unit (ICU) with neurosurgery consult. The initial exam revealed fixed and dilated pupils, suggestive of severe IH with brain herniation and the decision was made to proceed with an emergent intrathecal infusion with intraparenchymal intracranial pressure (ICP) monitoring, combined with EBP. A substantial clinical improvement was noted following the procedure. Within 45 minutes, the patient’s mental status improved to normal and pupillary dilation and areflexia were no longer observed. While the procedure may need to be repeated in cases of late deterioration, this report provides evidence that intrathecal bolus saline infusion with simultaneous ICP monitoring may be considered an effective measure to treat extreme cases of IH with associated brain herniation. If performed in a timely fashion, improvement of ICP numbers, and clinical resolution can be quite rapid.

## Introduction

Intracranial hypotension (IH) is commonly caused by a traumatic cerebrospinal fluid (CSF) leak secondary to lumbar puncture or surgery but may also arise spontaneously. IH is a relatively common condition associated with low CSF pressure, with the most common presentation of orthostatic headache. However, other symptoms and even neurological deficits may be present, including nausea, vomiting, diplopia, photophobia, hearing changes, ataxia, limb paresthesias, loss of bowel and bladder control, and changes in personality. Changes in the level of consciousness, including encephalopathy, stupor, and coma, may also occur [1-2]. 

The constellation of symptoms likely depends upon the degree of hypotension. As the CSF pressure decreases, the naturally buoyant force that suspends the brain is decreased, causing the brain to sag [2]. This may lead to crowding of the posterior fossa with downward displacement of the cerebellar tonsils that may be demonstrated on magnetic resonance imaging (MRI). Other MRI findings include diffuse pachymeningeal enhancement, decreased ventricular size, subdural fluid collections, and/or an enlarged pituitary gland [3-5]. Rest, hydration, and analgesia are often all that is needed to resolve an uncomplicated CSF hypotension headache; however, persistent cases may require an epidural blood patch (EBP) for resolution [6-8]. 

Herein, we present a particularly severe, life-threatening case of IH associated with headache and progressive neurological deterioration requiring intubation and mechanical ventilation. In order to achieve resolution, multiple epidural blood patches combined with intrathecal saline infusions were necessary.

## Case presentation

A 50-year-old male was originally admitted to an affiliate hospital after his wife witnessed him having convulsions at home. He had a history of intravenous (IV) drug abuse and was positive for human immunodeficiency virus (HIV), hepatitis B virus (HBV), and hepatitis C virus (HCV) antibodies. A lumbar puncture was performed, and he was found to have a CSF white blood cell count of 65 cells/mm^3^ with evidence of lymphocytic pleocytosis, suggesting the possibility of aseptic meningitis for which he was placed on Acyclovir.

**Figure 1:**
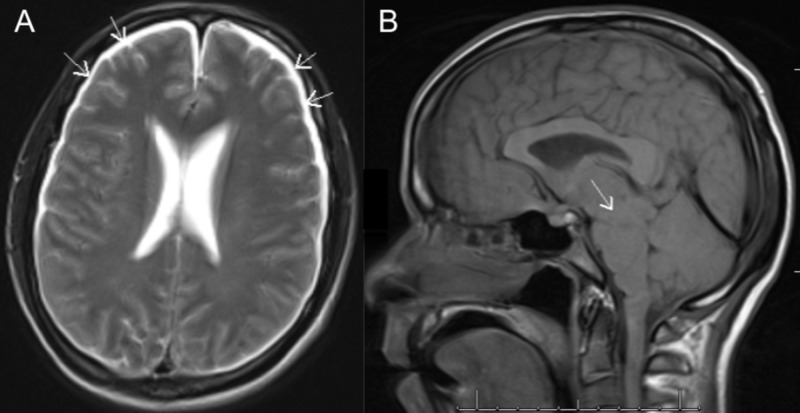
Initial brain magnetic resonance imaging (MRI) without contrast A) axial T2 images showing bilateral holohemispheric subdural effusions (arrows) with effacement of the sulci; B) sagittal T1 images, demonstrating distortion and downward displacement of the midbrain (arrow), most suggestive of cerebrospinal fluid (CSF) hypotension

Two days later, the patient developed a headache of 6/10 severity, not associated with nausea, vomiting, or visual changes. A brain MRI without contrast was performed, demonstrating new, bilateral holohemispheric subdural effusions with effacement of the sulci and distortion and downward displacement of the midbrain, most suggestive of interim development of CSF hypotension (Figure 1).

**Figure 2:**
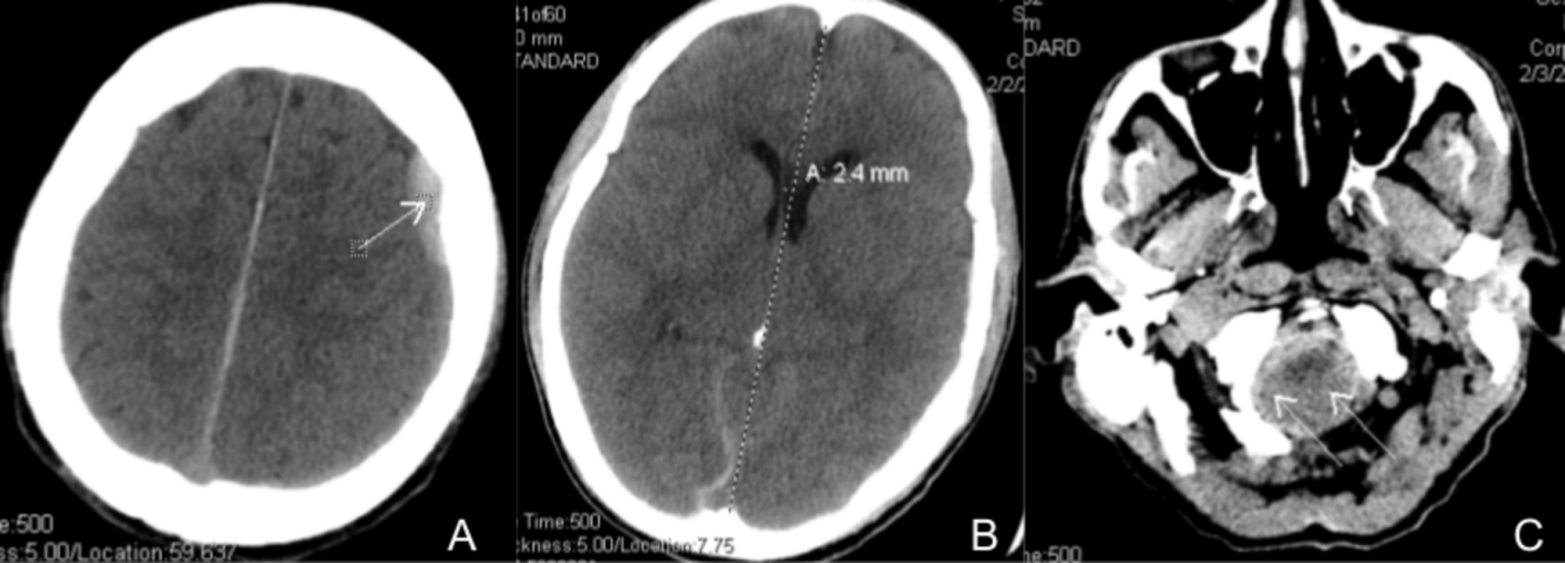
Brain computed tomography (CT) without contrast A) Worsening subdural hemorrhage (arrow); B) worsening mass effect with midline shift; C) low-lying cerebellar tonsils crowding the foramen magnum (double arrow)

Considering the complicated nature of his symptoms and findings, the patient was transferred to our primary institution to undergo EBP. Upon arrival, he was oriented to person and place only and complained of 10/10 headache, worse when upright, and localized to the frontal and temporal regions bilaterally. The patient’s examination was unremarkable, except for diffuse 4/5 weakness throughout all extremities with increased tone. A repeat brain computed tomography (CT) without contrast was performed. In comparison to the previous study, it showed worsening diffuse subdural hemorrhage, largest along the left frontal convexity with a 1 - 2 mm right midline shift and low-lying cerebellar tonsils (Figure 2). The planned blood patch was held on the day of admission as blood cultures grew out Staphylococcus aureus, which was ultimately found to be a contaminant, and the procedure was performed on the following day. The patients remained clinically stable periprocedurally, albeit without obvious improvement. 

The following day the patient developed an altered mental status and was sent for another stat CT scan of the brain that was read as stable compared to the previous study. While undergoing the scan, he reportedly developed agonizing respirations and was immediately intubated and admitted to the ICU. At this point, neurosurgery was consulted. Initial evaluation was limited due to intubation and sedation. Pupils were 5 mm and nonreactive bilaterally with no gaze preference and minimal corneal reflex. Minimal flexion of all extremities to noxious stimuli and decreased tone was also noted. The decision was then made to move forward with an emergent intrathecal saline infusion with intracranial pressure monitoring combined with an EBP. An intraparenchymal ICP bolt was inserted first at the bedside utilizing a routine sterile technique. The pressures were ranging between -7 and -13 mmHg. The patient was immediately turned to the lateral decubitus position and underwent placement of a lumbar spinal drain. While the ICP was monitored, a bolus of 163 ml of sterile saline was infused intrathecally via the drain until the ICP increased to 12 - 15 mmHg with good waveform. The spinal drain was subsequently clamped but left in place. A substantial clinical improvement followed the procedure. Within 45 minutes, the patient’s mental status improved to normal and pupillary dilation and areflexia were no longer observed. An hour after intrathecal infusion, he underwent a bedside EBP. 

On the following day, the patient appeared to be awake and interactive with pupils equal and reactive to light. The rest of the examination was unremarkable. The ICP remained within normal limits and it was felt to be safe to extubate the patient. He remained in the ICU for one more day with the clamped spinal drain and the ICP bolt in place until both were discontinued on ICU Day 3. Shortly thereafter, the patient’s mental status began to deteriorate again and the ICP monitor and lumbar drain were reinserted emergently without obtaining another set of neuroimaging. A second epidural blood patch and 45 ml normal saline intrathecal infusion were administered until there was a normalization of the ICP numbers. This time, the improvement back to baseline was instantaneous. A repeat CT of the brain was done after the procedures and still demonstrated persistent subdural fluid collections, effaced sulci, and low-lying cerebellar tonsils with normal ventricles and no midline shift.

On the following day, the patient was awake, alert, and oriented to person, place, and time. He continued to do well, and three days later, a more conservative approach was taken. The lumbar drain was removed first while leaving the ICP bolt in. The third EBP was done to prevent a possible continuous CSF leak after the removal of the relatively large-bore drain hardware. The ICP bolt was discontinued the following day after his ICP remained consistently within 8 to 12 mmHg range. A follow-up CT suggested a decrease in the anatomical evidence of CSF hypotension compared to the prior study with resolving subdural effusions, normal-appearing symmetric lateral ventricles, and greater CSF space surrounding the midbrain with decreased effacement. 

**Figure 3:**
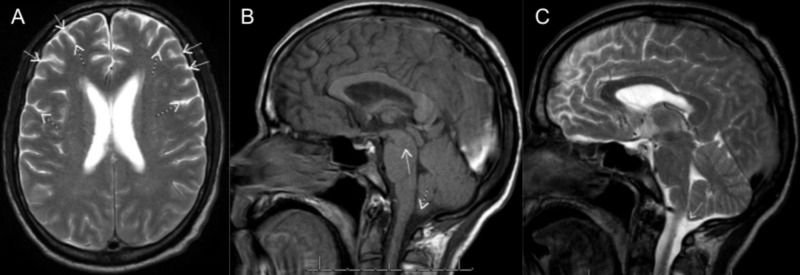
Post-treatment brain magnetic resonance imaging (MRI) with contrast A) Axial T2 image shows the resolution of subdural effusions (solid arrows) and normal cerebrospinal fluid (CSF) signal around the convexity sulci (dotted arrows); B) sagittal T1 image, shows a reversal of the downward displacement of the brainstem (solid arrow) and cerebellar tonsils (dotted arrow) no longer crowding the foramen magnum; C) sagittal T2 image illustrates the ascent of the cerebellar tonsils (solid arrow) and CSF signal around the caudal brainstem and dorsal cerebellum.

The patient continued to do well, and two days later a non-contrast MRI of the brain was performed. The previously noted downward herniation with effacement of the basal cisterns had resolved and the brainstem was no longer sagging, consistent with treatment of CSF hypotension (Figure 3). Bilateral subdural collections containing subacute hemorrhage still remained but were stable and felt to be non-surgical. Thoracic and lumbar spine MRI’s without contrast were also obtained, showing no primary or secondary findings of a CSF leak. The patient was subsequently discharged from the hospital doing well clinically and with a normal neurological examination.

## Discussion

This case outlines the therapeutic modalities available to treat severe intracranial hypotension with brain herniation as was evidenced by fixed and dilated pupils. Even though ICP monitoring and intrathecal infusions had been described in several previous case reports, extreme IH with brain herniation remains a very rare condition with poorly defined treatment principles. 

The vast majority of cases of CSF leakage causing headaches uncomplicated by other signs or symptoms may be treated conservatively with bed rest, hydration, and over-the-counter analgesics [7, 9]. Intravenous caffeine or theophylline has also been employed to relieve headaches [9]. More severe cases may require an EBP, such as the one described by Podkovik et al. where a patient with diffuse CSF leak throughout the cervicothoracic spine with a neurological decline was treated with bilateral subdural drain placement as a temporizing measure until definitive treatment with EBP was administered [10]. In our reported case, conservative management was largely supplanted for invasive therapy given the complicated nature of the presentation, with rapid neurologic deterioration supported by imaging. To move quickly in an attempt to reverse neurologic symptoms, a decision was made to combine an EBP with intrathecal saline infusion and continuous ICP monitoring as has been described in some reports [11-15]. Bolt placement for continuous ICP monitoring was parenchymal rather than a catheter to the ventricle, as it had to be placed very quickly. The ventricles were too small for a safe and quick ventriculostomy placement, and it was not felt that having a ventriculostomy in place would be of any advantage. Infusion into the ventricles was not considered a therapeutic option, as pushing saline into the ventricle in the context of brainstem herniation could potentially lead to non-communicating hydrocephalus secondary to compression of the cerebral aqueduct at the midbrain. This would worsen brain herniation due to increasing the pressure gradient across the foramen magnum. Therefore, injecting the fluid intrathecally from the lumbar spinal drain was considered a more physiological solution. 

Initially, the patient demonstrated rapid and marked improvement; however, following discontinuation of the intrathecal saline infusion, lumbar drains, and ICP monitoring, a rapid deterioration ensued. One possible explanation is that the first EBP was not sufficient to resolve the CSF leak and symptomatic improvement was primarily due to intrathecal saline infusion. It is well-documented that a single epidural blood patch might be sufficient to treat a CSF leak following lumbar puncture in the majority of patients. However, in the case of recurrent symptoms, it is recommended to repeat the procedure up to one or two more times [10]. Another possible explanation of the patient’s deterioration after EBP and saline infusion might be the removal of a relatively large-bore spinal drain tube, which could leave a hole in the dura, facilitating continuous postprocedural CSF leak and consequent IH. The authors felt that the solution to this problem could be the administration of another EBP immediately after drain removal and leaving the ICP bolt in place until the next day to document the successful resolution of hypotension. 

An intrathecal saline bolus infusion proved to be useful in the emergent treatment of patients with rapidly declining neurologic status and brain herniation [11-15]. Brainstem compression was evident in our patient with fixed, dilated pupils, minimal corneal reflex, and respiratory failure necessitating intubation and mechanical ventilation. In cases where the location of the CSF leak is unknown, such as in spontaneous intracranial hypotension, an intrathecal saline infusion may be considered a life-saving intervention while the location is sought [14-15]. Localizing the leak in refractory cases may allow for a more targeted EBP placement or surgical intervention. Looking to the future, the use of fibrin glue as an alternative to failed EBP is currently being investigated [12, 16]. Muram et al. also described a case where a patient experienced spontaneous IH with a decreasing level of consciousness who was successfully treated with intrathecal saline infusion, which bought time to locate the CSF leak within the thoracic spine and to surgically correct the defect [17]. 

## Conclusions

This report provides evidence that an intrathecal bolus saline infusion with simultaneous ICP monitoring may be considered an effective measure to treat extreme cases of IH, particularly those associated with brain herniation. If performed in a timely fashion, improvement of ICP numbers and a clinical resolution can be quite rapid. The procedure might need to be repeated in cases of late deterioration. EBP, on the other hand, plays a critical role in the treatment of the root cause of the problem (i.e., CSF leak). Multiple EBP administrations may be necessary as well before an effect takes hold. 
